# Th_1_-dominant cytokine responses in kidney patients after COVID-19 vaccination are associated with poor humoral responses

**DOI:** 10.1038/s41541-023-00664-4

**Published:** 2023-05-17

**Authors:** Yvette den Hartog, S. Reshwan K. Malahe, Wim J. R. Rietdijk, Marjolein Dieterich, Lennert Gommers, Daryl Geers, Susanne Bogers, Debbie van Baarle, Dimitri A. Diavatopoulos, A. Lianne Messchendorp, Renate G. van der Molen, Ester B. M. Remmerswaal, Frederike J. Bemelman, Ron T. Gansevoort, Luuk B. Hilbrands, Jan-Stephan Sanders, Corine H. GeurtsvanKessel, Marcia M. L. Kho, Marlies E. J. Reinders, Rory D. de Vries, Carla C. Baan, on behalf of RECOVAC Consortium

**Affiliations:** 1 Department of Internal Medicine, Nephrology and Transplantation, Erasmus MC Transplant Institute, University Medical Center, Rotterdam, The Netherlands; 2 Department of Hospital Pharmacy, University Medical Center, Rotterdam, The Netherlands; 3 Department of Viroscience, University Medical Center, Rotterdam, The Netherlands; 4 Department of Medical Microbiology and Infection Prevention, University Medical Center Groningen, Groningen, The Netherlands; 5 Center for Infectious Disease Control, National Institute for Public Health and the Environment, Bilthoven, The Netherlands; 6 Radboud Institute for Molecular Life Sciences, Department of Laboratory Medicine, Laboratory of Medical Immunology, Radboud University Medical Center Nijmegen, Nijmegen, The Netherlands; 7 Radboud Center for Infectious Diseases, Radboud University Medical Center Nijmegen, Nijmegen, The Netherlands; 8 Department of Internal Medicine, Division of Nephrology, University of Groningen, University Medical Center Groningen, Groningen, the Netherlands; 9 Department of Experimental Immunology, Amsterdam Infection and Immunity Institute, Amsterdam UMC, University of Amsterdam, Amsterdam, The Netherlands; 10 Renal Transplant Unit, Amsterdam UMC, University of Amsterdam, Amsterdam, The Netherlands; 11 Department of Nephrology, Radboud University Medical Center, Radboud Institute for Health Sciences, Nijmegen, The Netherlands

**Keywords:** RNA vaccines, Cellular immunity

## Abstract

Cytokines are regulators of the immune response against severe acute respiratory syndrome coronavirus-2 (SARS-CoV-2). However, the contribution of cytokine-secreting CD4^+^ and CD8^+^ memory T cells to the SARS-CoV-2-specific humoral immune response in immunocompromised kidney patients is unknown. Here, we profiled 12 cytokines after stimulation of whole blood obtained 28 days post second 100 μg mRNA-1273 vaccination with peptides covering the SARS-CoV-2 spike (S)-protein from patients with chronic kidney disease (CKD) stage 4/5, on dialysis, kidney transplant recipients (KTR), and healthy controls. Unsupervised hierarchical clustering analysis revealed two distinct vaccine-induced cytokine profiles. The first profile was characterized by high levels of T-helper (Th)_1_ (IL-2, TNF-α, and IFN-γ) and Th_2_ (IL-4, IL-5, IL-13) cytokines, and low levels of Th_17_ (IL-17A, IL-22) and Th_9_ (IL-9) cytokines. This cluster was dominated by patients with CKD, on dialysis, and healthy controls. In contrast, the second cytokine profile contained predominantly KTRs producing mainly Th_1_ cytokines upon re-stimulation, with lower levels or absence of Th_2_, Th_17_, and Th_9_ cytokines. Multivariate analyses indicated that a balanced memory T cell response with the production of Th_1_ and Th_2_ cytokines was associated with high levels of S1-specific binding and neutralizing antibodies mainly at 6 months after second vaccination. In conclusion, seroconversion is associated with the balanced production of cytokines by memory T cells. This emphasizes the importance of measuring multiple T cell cytokines to understand their influence on seroconversion and potentially gain more information about the protection induced by vaccine-induced memory T cells.

## Introduction

The coronavirus disease 2019 (COVID-19) pandemic, caused by the novel severe acute respiratory syndrome coronavirus-2 (SARS-CoV-2), still poses a significant health problem worldwide. In kidney disease patients, COVID-19 is associated with a three- to four-times increased risk of death compared to the general population^1^. This is mainly due to multiple comorbidities and their chronic immunosuppressive state, which is either dialysis-associated or therapy-mediated^2^. For this reason, kidney patients were prioritized by numerous health authorities around the world to be vaccinated with the mRNA-based COVID-19 vaccines: BNT162b2 (Pfizer/BioNTech) or mRNA-1273 (Moderna)^3,4^. Both are considered safe and effective in preventing severe COVID-19 in immunocompetent individuals^5–7^, dialysis patients, and patients suffering from chronic kidney disease (CKD)^2,8^. However, these vaccines proved poorly immunogenic in kidney transplant recipients (KTR), resulting in strongly reduced or even absent immune responses following the standard two-dose regimen of mRNA-based COVID-19 vaccines^2,9^. Multiple additional doses were shown to increase immunogenicity in KTR patients^8,10,11^.

Immunological correlates of protection have not yet been determined. Neutralizing antibodies are thought to play an important role^12^. However, it is known that spike (S)-specific antibodies after vaccination wane, and that newly emerging variants are antigenically distinct and can evade neutralizing antibody responses^13^. Cellular immune responses have also been implicated as a correlate of protection, and T cells thus far retain cross-reactivity with emerging variants^14^. It is therefore crucial to study the persistence of immune memory on the cellular level in more detail^9,15,16^. Cytokines produced by SARS-CoV-2-specific T cells also are important regulators of the magnitude, quality and course of the humoral response^17^. We speculate that the functionality of vaccine-induced SARS-CoV-2-specific T cells, partially reflected by their cytokine profiles, could correlate to the SARS-CoV-2-specific antibody response. Circulating levels of pro-inflammatory cytokines in plasma were shown to be increased in CKD and dialysis patients, while after kidney transplantation a decrease is observed resulting from an improvement in kidney function and the use of immunosuppressive medication^18,19^. For this reason, it is essential to investigate T cell cytokine profiles in response to SARS-CoV-2-specific stimulation instead of directly measuring cytokines in plasma.

In this study, we investigated the SARS-CoV-2 S-specific memory T cell cytokine response in patients with severe kidney damage due to CKD disease severity stage G4 or G5, patients on dialysis, kidney transplant recipients, and controls 28 days after the second vaccination with mRNA-1273. By performing unbiased and unsupervised analyses, we clustered these cytokine responses into specific profiles, and examined whether these profiles were associated with binding and neutralizing antibody levels.

## Results

### Baseline characteristics

A total of 212 participants were included as part of the RECOVAC immune response study at the Erasmus MC Rotterdam^2^ study site. Of these participants, 180 were eligible for inclusion in these analyses, including 42 healthy controls, 37 patients with CKD stage G4/5, 38 dialysis patients, and 63 KTRs (Supplemental Fig. 1). The baseline characteristics for each of these groups are presented in Supplemental Table 1. No differences among the groups were detected in S1-specific binding antibody levels and T cell responses at baseline (Supplemental Table 2).

### Two distinct vaccine-induced cytokine profiles were identified by unsupervised hierarchical clustering

**Fig. 1:**
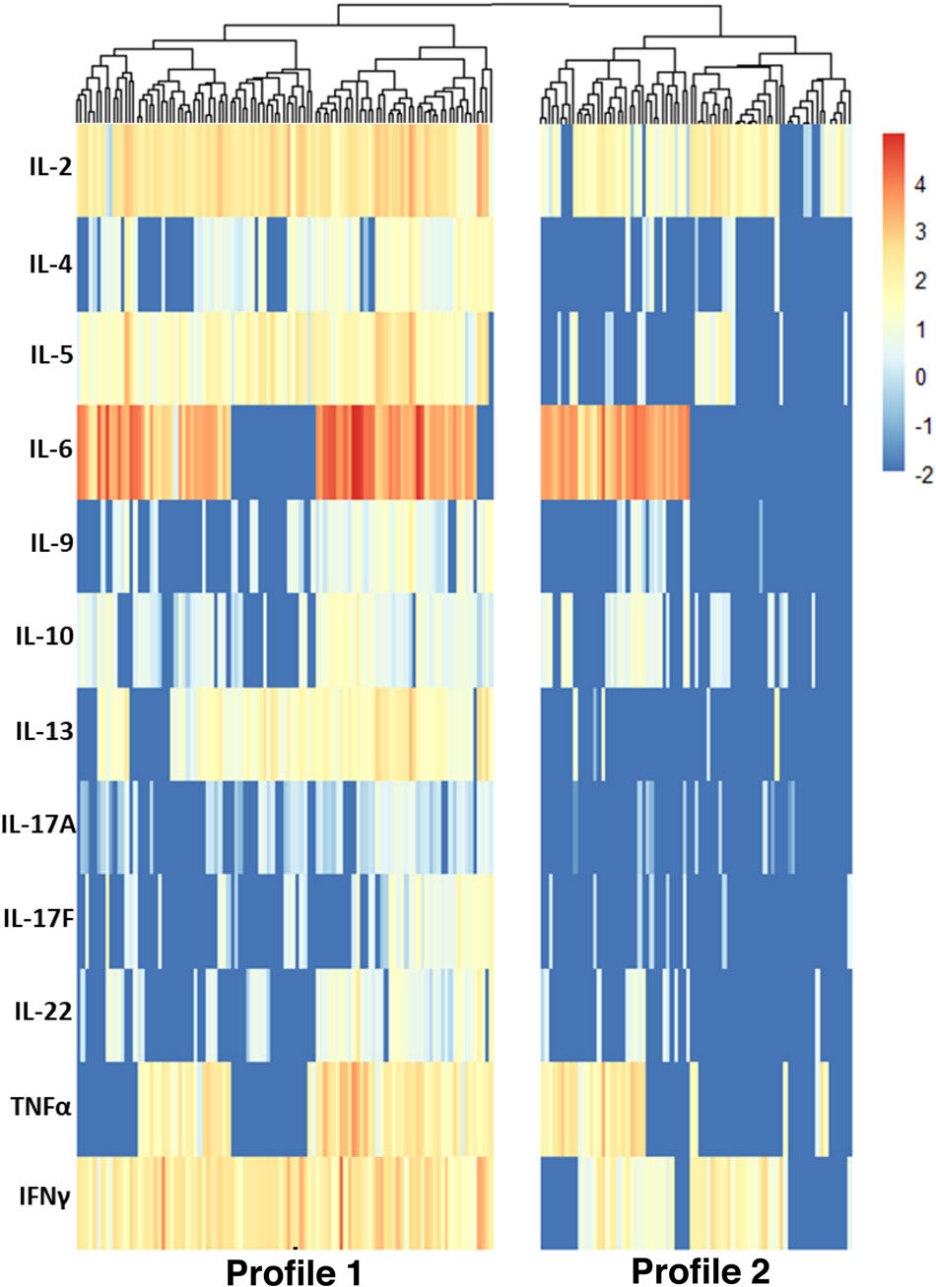
Unsupervised hierarchical clustering distinguishes two T cell cytokine profiles in study participants. Heatmap of the cluster analysis using log10-transformed z-scores. Red-to-blue color coding depicts log10 values. The top banner indicates cytokine profile cluster 1 and cluster 2.

We measured 12 different cytokines in plasma after stimulation of whole blood with overlapping S peptides. An unbiased and unsupervised data clustering analysis based on the T cell cytokine levels revealed two distinct cytokine profiles (Fig. 1). We determined the number of clusters based on the largest distance (dissimilarity) in the tree diagram. Baseline characteristics for each cytokine profile are presented in Table 1.

**Table 1:** Participant characteristics based on identified cytokine profiles.

Characteristic	Total (*N* = 180)	Cytokine profile 1 (*N* = 103)	Cytokine profile 2 (*N* = 77)	*P* value
Cohort, no. (%)				<0.01^c^
Control	42 (23)	31 (30)	11 14)	
CKD	37 (21)	23 (22)	14 (18)	
Dialysis	38 (21)	26 (25)	12 (16)	
KTR	63 (35)	23 (22)	40 (52)	
Sex, no. (%)				0.29^c^
Male	99 (55)	53 (51)	46 (60)	
Female	81 (45)	50 (49)	31 (40)	
Age at time of first dose, (IQR)—year	63.0 (52.8 to 71.0)	63.0 (49.0 to 70.0)	64.0 (54.0 to 72.0)	0.40^b^
BMI, (IQR)—kg/m^2^	26.9 (23.9 to 30.4)	26.5 (23.9 to 30.4)	27.3 (24.3 to 30.4)	0.35^b^
eGFR, (IQR)—mL/min/1.73 m^2^	33.3 (13.6 to 60.9)	30.7 (11.0 to 66.5)	35.3 (16.2 to 53.7)	0.88^b^
Leukocyte count, (IQR)—10^9^/L	7.6 (6.0 to 9.1)	7.4 (5.9 to 9.0)	7.6 (6.6 to 9.2)	0.42^b^
Lymphocyte count (IQR)—10^9^/L	1.7 (1.3 to 2.2)	1.9 (1.7 to 2.3)	1.5 (1.2 to 2.1)	<0.01^b^
Primary renal diagnosis, no. (%)				0.53^c^
Primary glomerulonephritis	16	7 (10)	9 (14)	
interstitial nephritis	1	0 (0)	1 (2)	
Familial/hereditary renal diseases	15	6 (8)	9 (14)	
Congenital diseases	6	2 (3)	4 (6)	
Vascular diseases	35	21 (29)	14 21)	
Secondary glomerular/systemic disease	1	1 (1)	1 (0)	
Diabetic kidney disease	17	10 (14)	7 (11)	
Other	31	19 (26)	12 (18)	
Dialysis characteristics				
Hemodialysis, no. (%)	–	18 (47)	9 (24)	
Peritoneal dialysis, no. (%)	–	8 (21)	2 (8)	
Time on dialysis (IQR)—mo		49.0 (16.0 to 189.0)	67.0 (29.0 to 162.3)	
Transplant characteristics	–			
First kidney transplant, no. (%)	–	20 (32)	32 51)	
Retransplantation, no. (%)		3 (5)	8 (13)	
Time after last transplantation (IQR)—year	–	8.5 (6.0 to 16.5)	4.1 (0.5 to 9.0)	
Last transplant				
Living, no. (%)	–	15 (24)	26 (41)	
Deceased, no. (%)	–	8 (13)	14 (22)	
Number of immunosuppressive agents (IQR)	–	0 (0 to 0)	1 (0 to 2)	
Immunosuppressive treatment				
Steroids, no. (%)	–	7 (11)	13 (21)	
Azathioprine	–	0 (0)	2 (3)	
Mycophenolate mofetil	–	19 (29)	31 49)	
Calcineurin inhibitor	–	21 (33)	39 (62)	
mTOR inhibitor	–	0 (0)	2 (2)	
Other	–	1 (2)	1 (2)	
Immune response on day 28 after the second vaccination				
S-specific binding antibodies (IQR)—BAU/ml^a^	3.09 (1.64 to 3.45)	3.27 (2.88 to 3.55)	2.54 (0.71 to 3.15)	
Neutralizing antibodies (IQR)—IU/ml^a^	2.42 (1.44 to 2.85)	2.71 (2.10 to 2.96)	1.86 (0.00 to 2.46)	<0.01^b^
IL-2, (IQR)—pg/ml^a^	2.13 (1.32 to 2.50)	2.41 (2.13 to 2.62)	1.36 (0.36 to 1.92)	<0.01^b^
IL-4, (IQR)—pg/ml^a^	–2.00 (–2.00 to 0.78)	0.65 (–2.00 to 1.17)	–2.00 (–2.00 to –2.00)	<0.01^b^
IL-5, (IQR)—pg/ml^a^	1.14 (–2.00 to 1.78)	1.60 (1.28 to 2.02)	–2.00 (–2.00 to –2.00)	<0.01^b^
IL-6 (IQR)—pg/ml^a^	2.99 (–2.00 to 3.60)	3.31 (1.21 to 3.66)	–2.00 (–2.00 to 3.41)	<0.01^b^
IL-9, (IQR)—pg/ml^a^	–2.00 (–2.00 to 0.51)	0.75 (0.20 to 0.75)	–2.00 (–2.00 to –2.00)	<0.01^b^
IL-10, (IQR)—pg/ml^a^	0.25 (–2.00 to 0.88)	0.49 (–049 to 0.88)	–2.00 (–2.00 to 0.57)	<0.01^b^
IL-13, (IQR)—pg/ml^a^	0.14 (–2.00 to 1.50)	1.36 (0.95 to 1.81)	–2.00 (–2.00 to –2.00)	<0.01^b^
IL-17A, (IQR)—pg/ml^a^	–2.00 (–2.00 to –0.21)	0.17 (–0.40 to 0.90)	–2.00 (–2.00 to 0.10)	<0.01^b^
IL-17F, (IQR)—pg/ml^a^	–2.00 (–2.00 to –0.07)	–2.00 (–2.00 to 0.67)	–2.00 (–2.00 to –2.00)	<0.01^b^
IL-22, (IQR)—pg/ml^a^	–2.00 (–2.00 to 0.51)	0.24 (–2.00 to 0.84)	–2.00 (–2.00 to –2.00)	<0.01^b^
TNF-α, (IQR)—pg/ml^a^	1.29 (–2.00 to 2.25)	1.63 (–2.00 to 2.32)	–2.00 (–2.00 to 1.93)	<0.01^b^
IFN-γ, (IQR)—pg/ml^a^	1.97 (1.07 to 2.44)	2.33 (1.90 to 2.59)	0.87 (–2.00 to 1.76)	<0.01^b^
Humoral immune responder, no. (%)				<0.01^b^
S1-specific binding antibodies 28 days after second vaccination	142 (79)	91 (88)	51 (66)	
S1-specific binding antibodies 6 months after second vaccination	141 (78)	91 (88)	50 (65)	<0.01^c^
Neutralizing antibodies 28 days after second vaccination	138 (77)	90 (87)	48 (62)	<0.01^c^
Neutralizing antibodies 6 months after second vaccination	142 (79)	91 (88)	51 (66)	<0.01^c^

*BMI* body mass index, *CKD* chronic kidney disease, *KTR* kidney transplant recipient, *eGFR* estimated glomerular filtration rate, *mTOR* mammalian target of rapamycin, *IL* interleukin, *mo* month.

Values are number (percentage) for categorical variables and median (interquartile range) for continuous variables.

^a^Median log10 transformed SARS-CoV-2-specific immune responses.

^b^*P* value based on nonparametric test (Kruskal–Wallis) test.

^c^*P* value based on Fisher’s exact test.

**Fig. 2:**
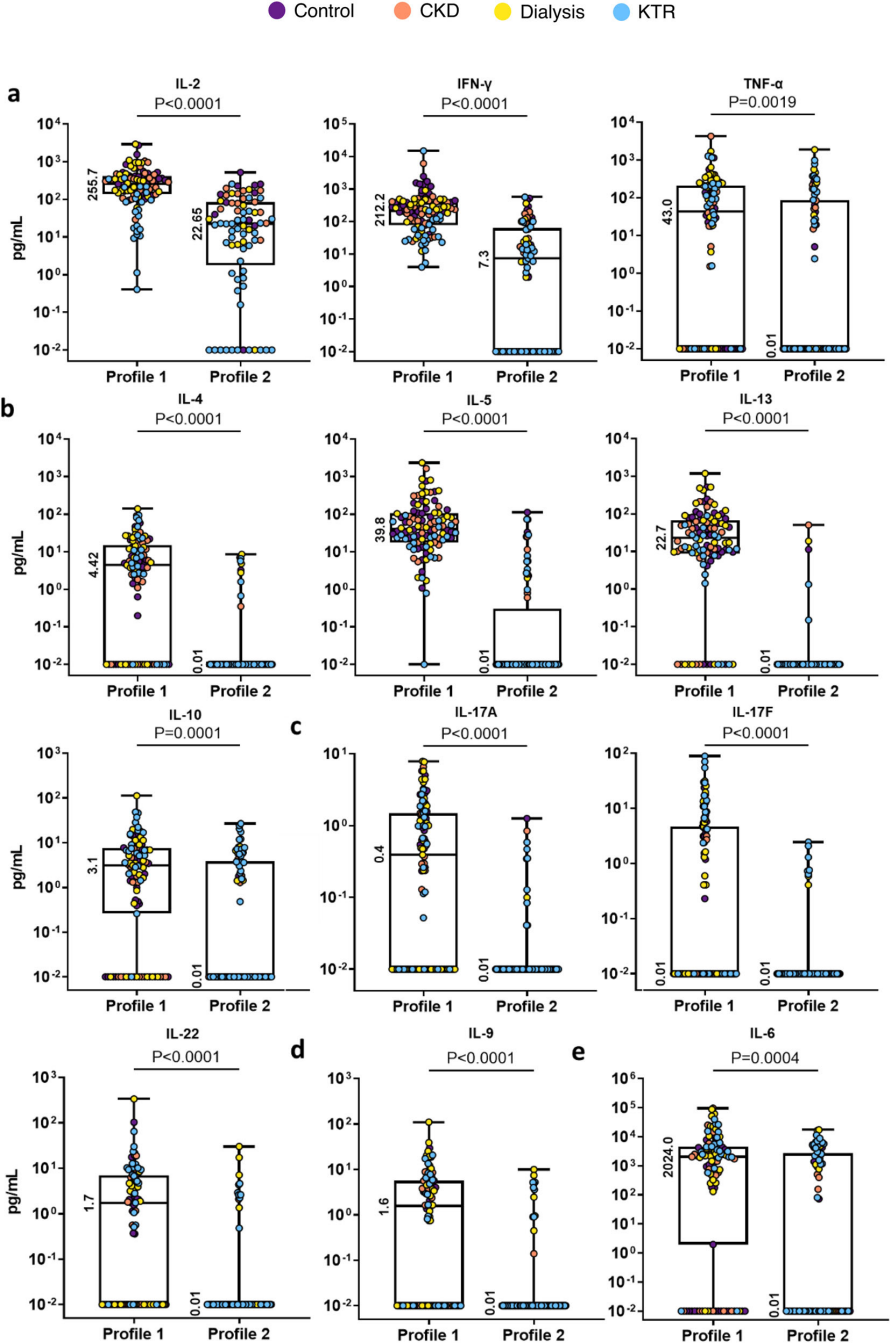
Vaccine-induced cytokine profiles have different cytokine levels. Cytokine levels depicted per cluster. **a** Levels of Th_1_ cytokines. **b** Levels of Th_2_ cytokines. **c** Levels of Th_17_ cytokines. **d** Levels of Th_9_ cytokine. **e** Levels of IL-6. The horizontal line and numbers within the whisker indicate the medians, and the tops and bottoms indicate the interquartile ranges. Mann–Whitney *U* tests were applied to the comparisons. Each symbol represents a participant.

In general, cytokine profile 1 was characterized by the presence of a strong T helper (Th)_1_ (IL-2, TNF-α, and IFN-γ) and Th_2_ cytokine response (IL-4, -5, -10, and -13). This was accompanied by low levels of Th_17_ (IL-17A, -22) and Th_9_ (IL-9) cytokines. In contrast, cytokine profile 2 was characterized by lower levels of IL-2 and IFN-γ. In addition, antigen-specific production of IL-4, -5, -6, -9, -10, -13, -17A, -17F, -22, and TNF-α in plasma after stimulation was detected in only a few participants with profile 2. In 2 out of 77 participants, none of the measured cytokines could be detected after specific T cell stimulation. Abundant production of IL-6 was identified in the majority of participants with either profile, and few participants produced IL-17F. The results of these analyses are presented in Fig. 2; the immune responses per study cohort are shown in Supplementary Table 2.

Whereas the two profiles differed significantly in cytokine levels, they also differed in the representation of the different participant subgroups. The Th_1_ and Th_2_-producing cytokine profile 1 contained relatively more controls (31 of 42 participants), CKD G4/5 patients (23 of 37 participants), and patients on dialysis (26 of 38 participants), while Th_1_-dominated cytokine profile 2 contained relatively more KTR (40 of 63 participants). Moreover, there were significant differences between participants with distinct profiles in terms of lymphocyte count, transplant characteristics, and use of immunosuppressive drugs (Table 1). Lymphocyte counts were significantly higher, the proportion of participants with a first kidney transplant was higher, and time after transplantation was longer in participants with cytokine profile 1. Regarding immunosuppressive drugs, the percentage of patients on steroids, mycophenolate mofetil, and calcineurin inhibitors was higher in the Th_1_-dominated cytokine profile 2 than in the Th_1_ and Th_2_-producing cytokine profile 1. Sex and age distribution did not differ significantly between the two cytokine profiles.

### Vaccine-induced T cell cytokine profiles are associated with S1-specific binding antibodies and neutralizing antibodies

**Fig. 3:**
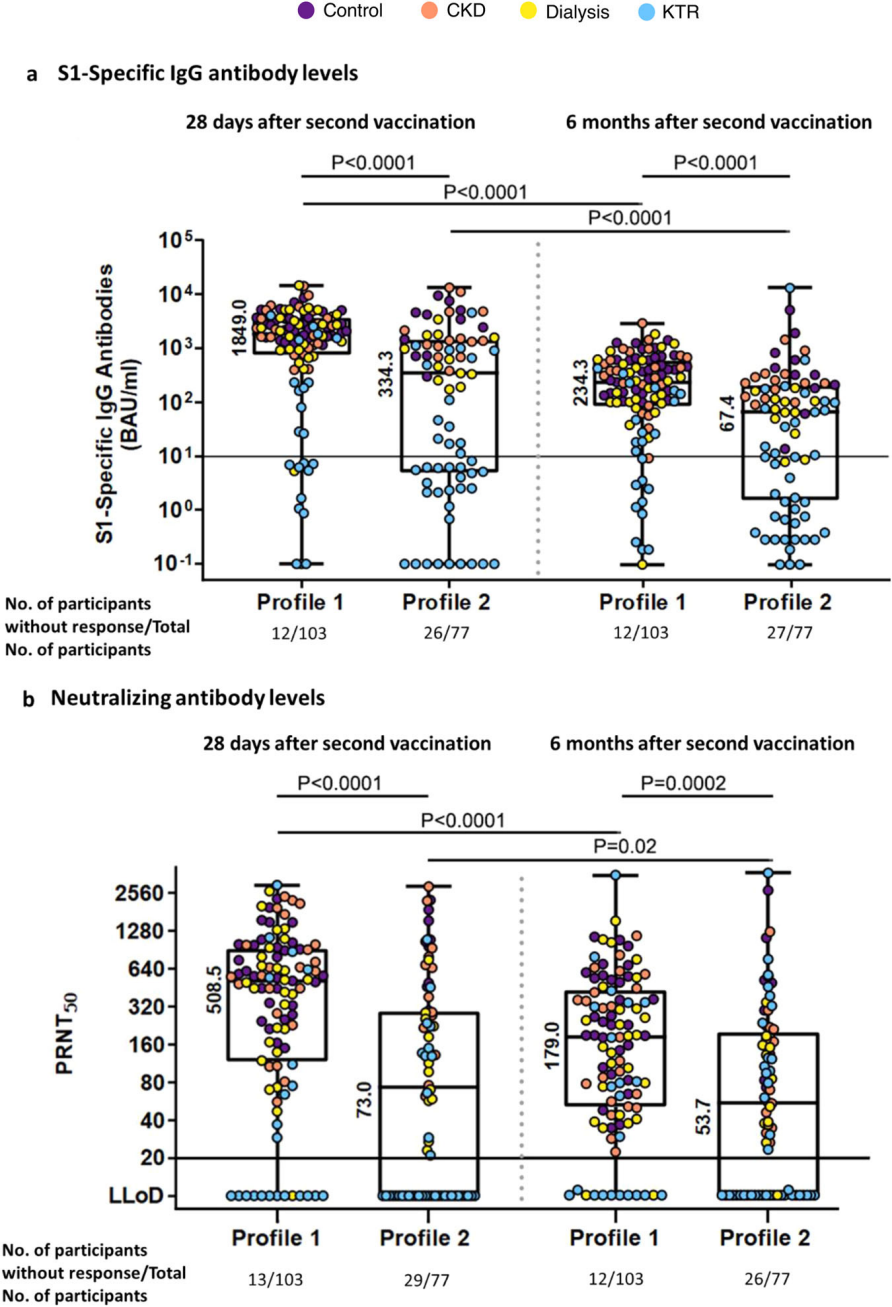
Vaccine-induced cytokine profiles are associated with SARS-CoV-2 antibody responses and kinetics. **a** Levels of SARS-CoV-2 spike protein 1 (S1)-specific IgG antibodies at 28 days and 6 months after the second vaccination depicted per cluster. The cutoff value for a response was set at 10 binding antibody units (BAU) per milliliter (solid horizontal line). **b** Levels of SARS-CoV-2-specific neutralizing antibodies at 28 days and 6 months after the second vaccination. The cutoff value for a vaccine response was a PRNT50 of 20 (solid horizontal line). All data are presented in box-and-whisker plots. The horizontal line and number within the whisker indicate the medians and the tops and bottoms indicate the interquartile ranges. Mann–Whitney *U* tests were applied to compare the waning over the study period between profiles. Wilcoxon signed-rank tests were applied to compare the waning within one profile over the study period. Each symbol represents a participant.

We next investigated whether the distinct cytokine profiles were associated with S1-specific binding and neutralizing antibody levels after the second vaccination. At 28 days after second vaccination, based on the responder criteria, 91 of 103 (88%) participants with cytokine profile 1 had detectable S1-specific binding antibodies, and 90 (87%) of these 103 participants had detectable neutralizing antibodies (Table 1 and Fig. 3). Noteworthy is that participants with this cytokine profile who did not have binding and neutralizing antibodies were mainly KTR participants. Of the 23 KTRs in cytokine profile 1, 11 (48%) had no humoral response. Of the participants with cytokine profile 2, only 51 of 77 (66%) participants developed detectable S1-specific binding antibodies, and of these 77 participants 48 (62%) had detectable neutralizing antibodies (Fig. 3).

The S1-specific binding antibody levels were significantly lower in cytokine profile 2 as compared to cytokine profile 1 (*P* < 0.0001) at 28 days after the second vaccination (Fig. 3a). Similarly, the neutralizing antibody levels were significantly lower in cytokine profile 2 as compared to cytokine profile 1 (*P* < 0.0001, Fig. 3b). A multivariate quantile regression (controlling for original study cohort) showed that the trends were similar but statistical significance disappeared after bootstrapping for the association between cytokine profile and S1-specific binding antibodies (beta −0.24; 95% CI −0.54 to −0.05; *P* = 0.09), and neutralizing antibodies (beta −0.20; 95% CI = −0.49 to 0.09; *P* = 0.20) (Table 2). Although the estimates can be significant (the 95% CI does not contain 0), after bootstrapping (simulating) the statistical significance may be altered.

**Table 2:** Multivariate quantile regression on log_10_-transformed S1-specific binding and neutralizing antibodies 28 days and 6 months after the second mRNA-1273 vaccination.

Variable or covariate	Reference category	Beta S1-specific binding antibodies 28 days	Beta S1-specific binding antibodies 6 months	Beta PRNT_50_ 28 days	Beta PRNT_50_ 6 months
Cytokine profile 2	Cytokine profile 1	–0.24 (–0.54 to –0.05) *P* = 0.09	–0.29 (–0.48 to –0.10) *P* < 0.01	–0.20 (–0.49 to 0.09) *P* = 0.20	–0.40 (–0.77 to –0.11) *P* = 0.04
CKD	Control	–0.06 (–0.28 to 0.19) *P* = 0.66	0.03 (–0.27 to 0.11) *P* = 0.79	–0.08 (–0.27 to 0.21) *P* = 0.51	0.07 (–0.54 to 0.28) *P* = 0.75
Dialysis	Control	–0.20 (–0.52 to 0.08) *P* = 0.16	–0.45 (–0.70 to –0.21) *P* < 0.01	–0.31 (–0.63 to –0.05) *P* = 0.04	–0.24 (–0.54 to 0.01) *P* = 0.14
KTR	Control	–2.45 (–2.71 to –1.96) *P* < 0.01	–1.66 (–2.30 to –1.29) *P* < 0.01	–2.66 (–2.96 to –2.42) *P* < 0.01	–1.38 (–2.40 to –0.53) *P* = 0.04

*CKD* chronic kidney disease, *KTR* kidney transplant recipient.

Outcome variable: Log_10_-transformed titer value of S-specific binding antibodies and PRNT_50_. All *P* values are based on the bootstrapping procedure.

At 6 months after second vaccination, 91 of 103 participants (88%) with Th_1_ and Th_2_ producing cytokine profile 1 had detectable S1-specific binding antibodies and neutralizing antibodies. In participants with the Th_1_-dominated cytokine profile 2, 50 (65%) of 77 participants had detectable S1-specific binding antibodies and 51 (66%) of 77 participants had detectable neutralizing antibodies 6 months after the second vaccination (see Fig. 3).

The S1-specific binding antibody levels were significantly lower in cytokine profile 2 as compared to cytokine profile 1 (*P* < 0.0001) at 6 months after the second vaccination (Fig. 3a). Similarly, the neutralizing antibody levels were significantly lower in cytokine profile 2 as compared to cytokine profile 1 (*P* < 0.002, Fig. 3b). A multivariate quantile regression (controlling for original study cohort group) showed that the trends were similar and statistical significance remained for the association between cytokine profile and S1-binding antibodies (beta −0.29; 95% CI −0.48 to −0.10; *P* < 0.01), and neutralizing antibodies (beta −0.40; 95% CI −0.77 to −0.11; *P* = 0.04) both measured 6 months after the second vaccination (Table 2).

### S1-specific binding and neutralizing antibodies

We correlated the S1-specific binding and neutralizing antibodies at 28 days and 6 months after the second vaccination. A positive significant correlation between levels of S1-binding antibodies and neutralizing antibodies in serum samples was found at 28 days after the second vaccination (Spearman’s rank correlation 0.90; *P* < 0.0001). Similarly, we found a significant and positive correlation between S1-binding antibodies and neutralizing antibodies at 6 months (Spearman’s rank correlation coefficient of Spearman’s rank correlation 0.78; *P* < 0.0001, see Supplemental Fig. 3).

### Waning of S1-specific binding and neutralizing antibodies in participants with different vaccine-induced cytokine profiles

Waning of antibodies was observed in all participants 6 months after the primary vaccine series. With Th_1_ and Th_2_ producing cytokine profile 1, S1-specific binding antibodies decreased significantly between 28 days and 6 months after vaccination, with a 6.6-fold reduction (*P* < 0.0001; see Fig. 3a). A similar waning was observed in participants with the Th_1_-dominated cytokine profile 2, with a 5.2-fold reduction (*P* < 0.0001; Fig. 3a). The waning (fold reduction) in cytokine profile 1 as compared to cytokine profile 2 was significantly higher (*P* = 0.045). Neutralizing antibody levels waned significantly in participants with cytokine profile 1, 2.0-fold reduction (*P* < 0.0001; see Fig. 3b). In addition, we found waning in participants with cytokine profile 2 (1.00-fold reduction; *P* = 0.02; see Fig. 3b). The waning (fold reduction) in cytokine profile 1 was significantly higher compared to cytokine profile 2 (*P* < 0.002).

### IL-2 levels are a sensitive readout parameter for T cell responses

Based on the production of IFN-γ after stimulation of whole blood, 85% of the study participants were identified as T cell responders, while 93% of the participants were identified as responders based on IL-2 production. This was especially apparent in KTR, in which 67% was identified as T cell responder based on IFN-γ levels, and 84% based on IL-2 levels. A positive correlation between Th_1_ cytokine levels of IFN-γ and IL-2 was found (Spearman’s rank correlation coefficient of 0.82, *P* < 0.001; see Supplemental Fig. 4).

## Discussion

Our study aimed to define the relationship between SARS-CoV-2-specific memory T cell cytokine profiles and antibody responses. We investigated cytokine profiles after stimulation of whole blood obtained 28 days after mRNA-1273 vaccination of kidney patients and controls. Using an unbiased clustering analysis, which was performed on SARS-CoV-2-specific cytokine production 28 days after the second vaccination, we were able to identify two distinct profiles. A balanced Th_1_ and Th_2_ T cell cytokine response (cytokine profile 1) was associated with higher levels of S1-specific binding antibodies and neutralizing antibodies at 28 days and 6 months after the second vaccination than a profile in which the Th_1_ T cell cytokines dominated (cytokine profile 2). Also, our study revealed that to determine whether kidney patients have a T cell response, detection of IL-2 after specific stimulation is more sensitive than IFN-γ.

Newly emerging SARS-CoV-2 variants are antigenically distinct and partially escape neutralizing antibodies^20^. In contrast, SARS-CoV-2-specific memory T cells have retained cross-reactivity with emerging variants^21,22^. Since the newer variants from the Omicron sub-lineage cause relatively mild disease, this could indicate that the protective role of the SARS-CoV-2-specific memory T cell responses is becoming more important. T cell responses against SARS-CoV-2 are often identified by the production of a single effector cytokine, namely the Th_1_ cytokine IFN-γ^2,22–24^. Different approaches are used for detection of this cytokine, including IFN-γ release assay (IGRA), IFN-γ ELISpot, or intracellular cytokine staining. Here, we show that measuring IL-2 could be a more sensitive alternative readout to identify kidney patients with a T cell response. A disadvantage of these approaches is that T cell subsets producing other cytokines could be missed, and therefore the T cell response after infection or vaccination can be underestimated. We identified two distinct cytokine profiles by measuring 12 cytokines after specific stimulation; the first was characterized by high levels of Th_1_ (IL-2, TNF-α, and IFN-γ) and Th_2_ (IL-4, -5, -10, and -13) cytokines, and to a lower extent Th_17_ (IL-17A, -22) and Th_9_ (IL-9) cytokines. The second cytokine profile was characterized by low production levels of Th_1_ cytokines and nearly the absence of Th_2_ cytokine production.

Th_1_ cells promote cellular immunity, which is essential in host defense against intracellular pathogens such as viruses^25,26^, whereas Th_2_ cells mediate the activation and maintenance of the humoral immune response^25,26^. A balanced Th_1_/Th_2_ response is therefore crucial. For example, Th_2_-biased responses are associated with vaccine-associated enhanced respiratory disease, as reported for measles and respiratory syncytial virus infection^27,28^. In our study, higher antibody levels were associated with combined Th_1_ and Th_2_ production (cytokine profile 1), demonstrating that the production of S-specific antibodies is enhanced by balanced T cell responses.

We show that the number of immunosuppressive drugs that was used affected vaccine-induced T cell responses, with a higher percentage of patients on immunosuppression in cytokine profile 2. It is known that patients who are treated with mycophenolate mofetil (MMF) mount lower SARS-CoV-2-specific cellular and humoral responses^2,8,9^. This is because the active metabolite of MMF, mycophenolic acid, inhibits lymphocyte proliferation^29^. Calcineurin inhibitors inhibit the production of T cell cytokines (IL-2, TNF-α, IFN-γ, IL-4) and subsequently impair the efficient formation of humoral immune responses^30,31^. Another factor associated with reduced cytokine diversity is time since transplantation^8,32,33^. In our study, KTR with T cells producing Th_1_ and Th_2_ cytokines were longer after transplantation in comparison to KTR with the Th_1_ dominant cytokine profile. We speculate that this is the effect of less intense immunosuppressive drug therapy.

It is important to emphasize that we identified KTR participants with cytokine profile 1 who had a functional T cell response in the absence of a detectable humoral response. It is already known that exposure to SARS-CoV-2 can induce virus-specific T cells without seroconversion^34^. On the other hand, we identified participants with cytokine profile 2 who had a less functional T cell response, but had detectable humoral immune responses. We speculate that this may be because of the presence of another less diverse T cell cytokine profile, that is associated with a good humoral immune response.

Impaired vaccine responses to other vaccines in CKD and dialysis patients, such as the inactivated influenza vaccine and subunit hepatitis B vaccine, are well described^35,36^. Strikingly, in our study, the vast majority of CKD and dialysis patients seroconverted and developed functional T cell responses after vaccination. We speculate that mRNA-based vaccination induces a functional response in these patients, because the S protein is endogenously produced and subsequently processed and presented to CD8+ cytotoxic T cells^37–39^. Via this pathway, training of the immune system is not solely dependent on antigen presentation by professional antigen-presenting cells that are known to be less present and have altered functions in these patient groups^40,41^ As mRNA-based COVID-19 vaccines can effectively induce cellular and humoral immune responses in CKD patients and patients on dialysis, it could be of interest to pursue the development of mRNA-based vaccines against other diseases for immunocompromised patients.

Our study also has limitations; first, the sample size does not allow studying differences in clinical efficacy against infection or disease between the two identified cytokine profiles. Second, the included population is heterogeneous (ranging from healthy controls to individuals with severely impaired kidney function and transplanted kidneys). This may impact the generalizability of the results. For future research, it is important to examine the correlations of cytokine profiles and humoral immune responses in other cohort studies.

In conclusion, SARS-CoV-2-specific memory T cells are present in a large number of kidney patients and healthy individuals 28 days after the second mRNA-1273 vaccination. Seroconversion was shown to be associated with the pattern and balance of cytokines produced by these memory T cells. This emphasizes the importance of measuring multiple T cell cytokines, instead of one, to gain more information about the protection induced by these vaccine-induced memory T cells and their influence on seroconversion. The classification of participants by SARS-CoV-2-specific T cell cytokine profiles may guide personalized vaccination and therapeutic strategies such as monoclonal antibody therapies.

## Methods

### Participants and COVID-19 vaccination

The SARS-CoV-2-specific memory T cell cytokines were measured in 180 participants of the multicenter RECOVAC IR study, who were enrolled at the Erasmus MC Rotterdam^2^. Further, inclusion and exclusion criteria are reported in Supplemental Fig. 1. The RECOVAC IR study was approved by the Dutch Central Committee on Research Involving Human Subjects (CCMO, NL76215.042.21) and the institutional review board of the Erasmus MC Rotterdam (MEC2020-662), and registered at clinicaltrials.gov (NCT04741386). Written informed consent was obtained from all participants. Four different cohorts were included; cohort A: participants without kidney disease (*n* = 42; control group, eGFR >45 mL/min/1.73 m^2^); Cohort B: patients with CKD stage G4/5 (*n* = 44; eGFR <30 mL/min/1.73m^2^); cohort C: patients undergoing hemo- or peritoneal dialysis (*n* = 44); and cohort D: KTR (*n* = 74). All participants received two doses of the mRNA-1273 COVID-19 vaccine (100 µg; Moderna Biotech Spain, S.L.) with an interval of 28 days^2^. Whole blood samples were obtained before vaccination (baseline), and at 28 days and 6 months after the second vaccination. The samples were processed within 12 h of the blood draw. For clarity of the study design, we made an infographic providing accessible visual information about the study in Supplemental Fig. 2.

### T cell cytokines

The SARS-CoV-2-specific T cell response was measured at baseline and 28 days after the second vaccination, using the commercially available IFN-γ Release Assay (IGRA, QuantiFERON, QIAGEN, Hilden, Germany) in heparinized whole blood as described previously^2^. Briefly, SARS-CoV-2 antigen tubes containing overlapping peptides representing the S protein and stimulating both CD4^+^ and CD8^+^ T cells (Ag2) were incubated with freshly heparinized whole blood for 20–24 h at 37 °C. After incubation, plasma was collected and frozen until analysis of the IFN-γ response that was published by Sanders et al. by enzyme-linked immunosorbent assay (ELISA)^2^. After several weeks, plasma samples were transferred to –80 °C, until measuring additional cytokines. The cytokines (interleukin (IL)-2, 4, 5, 6, 9, 10, 13, 17A, 17F, 22, IFN-γ, and TNF-α) present in the plasma of ex vivo 20–24 h stimulated whole blood samples were measured using a human Th cytokine panel (12- plex) kit (LEGENDplex, Biolegend, CA, USA). Briefly, after thawing on ice, plasma samples were centrifuged at 1000×*g* for 10 min at room temperature. Twofold dilutions were prepared and incubated for 2 h with monoclonal capture antibody-coated beads. Subsequently, the beads were washed and incubated for one hour with biotin-labeled detection antibodies and finally incubated with streptavidin-PE for 30 min. After staining, beads were acquired by flow cytometry on a BD FACSCanto™ II with BD FACSDiva™ software (BD Bioscience, NJ, USA). The data obtained was analyzed with LEGENDplex V8.0 software (BioLegend). The quantity of each respective cytokine was calculated based on the intensity of the streptavidin-PE signal and a freshly prepared standard curve. Results were expressed in picogram cytokine/mL after subtraction of the NIL control value. Samples that had a negative value after subtraction were set at 0.01 picogram/mL (pg/mL). As an internal quality control for the cytokine measurements, we performed Spearman’s correlation analysis on the IFN-γ concentrations of the same samples measured by both ELISA (data presented in the original publication^2^) and multiplex bead assay, and found that these were highly correlated (see Supplemental Fig. 5). These analyses excluded participants with a negative response in one of the IFN-γ measurements. In addition, the Bland–Altman agreement analysis showed that there is some degree of bias (bias = 2.30, 95% limits of agreement 1.16–3.45), suggesting that Legendplex gives higher IFN-γ values (see Supplemental Fig. 5) compared to the commercial ELISA.

### SARS-CoV-2 S1-specific IgG binding antibodies

SARS-CoV-2 S1-specific IgG binding antibodies were measured in serum at baseline, 28 days and 6 months after the second vaccination by a validated fluorescent bead-based multiplex-immunoassay^2^. The specificity and sensitivity of the assay are 99.7% and 91.6%, respectively, and were determined and described previously^42^. Concentrations were interpolated from a reference consisting of pooled sera using a five-parameter logistic fit and the National Institute for Biological Standards and Control/World Health Organization (NIBSC/WHO) COVID-19 reference serum 20/136, and expressed as international binding antibody units per mL (BAU/mL). The cutoff value for positivity was considered at ≥10 BAU/mL S1-specific binding antibodies based on previous publications^2,21,42^.

### Detection of virus-neutralizing antibodies by plaque reduction neutralization assay

Neutralizing antibodies against the ancestral D614G SARS-CoV-2 were tested 28 days and 6 months after the second vaccination by plaque reduction neutralization test (PRNT_50_) on Vero-E6 cells (ATCC) as described previously^21^. Briefly, heat-inactivated sera were twofold diluted in Opti-MEM medium starting at a dilution of 1:10 in 60 μl. 60 μl of SARS-CoV-2 virus suspension was added to each well and incubated at 37 °C for 1 h (leading to ±1000 plaques per well in infection controls). After 1 h of incubation, the virus-antibody mixtures were transferred on to the Vero-E6 cells and incubated for 8 h. Subsequently, cells and plaques were fixed with 10% formaldehyde and plaques were stained with polyclonal rabbit anti–SARS-CoV-2 nucleocapsid antibody (Sino Biological; 40143-V08B) and a secondary peroxidase-labeled goat anti-rabbit IgG (Dako; P0448). Plates were developed with 3,3′,5,5′-tetramethylbenzidine substrate (TrueBlue; Kirkegaard & Perry Laboratories) and the number of infected cells per well was counted by using an ImmunoSpot Image Analyzer (CTL Europe GmbH). The dilution that would yield 50% reduction of plaques (PRNT50) compared with the infection control (included on all plates) was evaluated by determining the proportionate distance between two dilutions from which an endpoint titer was calculated. The cutoff value for positivity was considered at PRNT50 ≥ 20 based on assay validation.

### Statistical analysis

We analyzed the data in four steps. First, as we collected samples from the Erasmus MC Rotterdam participants in the RECOVAC Immune Response study exclusively (see Supplemental Fig. 1), the baseline characteristics of each group were presented^2^. Categorical variables are presented as numbers (percentages), and group differences were tested by Fisher’s exact test. Continuous variables are presented as median (interquartile ranges). Between median group differences were tested by Kruskall–Wallis test (for four groups).

Second, cytokine values at 28 days after the second vaccination were log_10_-transformed. Unbiased and unsupervised hierarchical clustering (using Euclidian distance) was performed on the log_10_-transformed cytokine values. The optimal number of clusters was assigned using the R package NbClust. The number of clusters was determined based on the largest distance in the tree diagram based on dissimilarity^43^. Subsequently, a heatmap was plotted using the R package pheatmap (V1.0.12). Furthermore, the 28 days after vaccination, important clinical characteristics of each identified cluster were calculated in a similar fashion to the baseline characteristics.

Third, to assess the association between the cytokine clusters as found in the previous step, we presented the effective levels of the S1-specific binding antibodies and neutralizing antibodies per cytokine cluster. Pair-wise comparison of log_10_-transformed S1-specific binding antibodies and neutralizing antibodies between the two identified cytokine clusters was performed by a Mann–Whitney *U* test. Also, we performed multivariate quantile regression on the log_10_-transformed S1-specific binding antibodies levels at 28 days and 6 months after second vaccination, with cytokine profile and original group as covariates. For multivariate regression, we presented the beta’s and 95% confidence intervals (95% CIs). Standard errors and 95% CIs are estimated using the bootstrapping methods.

Fourth, to examine the association between S1-specific binding antibodies and neutralizing antibodies at 28 days and 6 months after the second vaccination, the Spearman’s correlation coefficient was used. In addition, waning (fold reduction) over the study period and per cytokine profile (6 months versus 28 days after vaccination) is done using a Mann–Whitney *U* test (between profiles, two independent samples), Wilcoxon signed-rank test (within one profile, similar patients measured over the study period). In addition, we tested the association between Th_1_ cytokine IL-2 and IFN-γ using Spearman’s correlation coefficient. Also, we examined whether the responder rate as measured by Th_1_ cytokine IL-2 and IFN-γ. A patient was a responder when the response was >0.01 pg/ml for both cytokines. Further, we also examined the correlation and agreement between the measurement of IFN-γ levels 28 days after the second vaccination with Legendplex versus those measured using ELISA. Statistical analyses were carried out with GraphPad Prism software version 9.1.2, and Rstudio software version 4.0.5. *P* values < 0.05 were considered statistically significant.

### Reporting summary

Further information on research design is available in the Nature Research Reporting Summary linked to this article.

## Supplementary information

Supplementary File

REPORTING SUMMARY

## Data Availability

All data used to support the findings of this study are available from the corresponding author upon reasonable request.

## Code availability

Please contact the corresponding author to obtain the R code used in this study.
