# Supplementary material for: Th1-dominant cytokine responses in kidney patients after COVID-19 vaccination are associated with poor humoral responses
Source: NPJ Vaccines. 2023 May 17;8:70. doi: 10.1038/s41541-023-00664-4 (PMC10191401; doi:10.1038/s41541-023-00664-4)
Supplement: Supplementary file 1 — Supplementary File [file 41541_2023_664_MOESM1_ESM.pdf]

Supplementary File for "Th1-dominant Cytokine Responses in Kidney Patients After COVID-19 Vaccination are Associated with Poor Humoral Responses" by den Hartog et al.

RECOVAC Consortium

Affiliation	Team Member
Department of Nephrology and Hypertension, University Medical Center Utrecht, Utrecht, The Netherlands	Alferso C. Abrahams
Department of Nephrology, Radboud University Medical Center, Radboud Institute for Health Sciences, Nijmegen, The Netherlands	Marije C. Baas
Department of Internal Medicine, division of Nephrology, Maastricht University Medical Center and CARIM school for cardiovascular disease, University of Maastricht, Maastricht, The Netherlands	Pim Bouwmans, Marc H. Hemmelder
Dutch Registry RENINE, Nefrovisie, Utrecht, The Netherlands	Marc A.G.J. ten Dam
Renal Transplant Unit, Amsterdam UMC, University of Amsterdam, Amsterdam, The Netherlands	Sophie C. Frölke, Dorien Standaar
Department of Medical Microbiology and Infection Prevention, University Medical Center Groningen, Groningen, The Netherlands	Marieke van der Heiden, Celine Imhof*
Department of Internal Medicine, Division of Nephrology, University of Groningen, University Medical Center Groningen, Groningen, the Netherlands	Priya Vart, Yvonne M.R. Adema
Department of Internal Medicine, Nephrology and Transplantation, Erasmus MC Transplant Institute, Erasmus Medical Center, Rotterdam, The Netherlands	Marieken J. Boer-Verschragen, Wouter B. Mattheussens, Ria Philipsen
Department Viroscience, Erasmus Medical Center, Rotterdam, the Netherlands	Marion P.G. Koopmans, Djenolan van Mourik
Center for Infectious Disease Control, National Institute for Public Health and the Environment, Bilthoven, the Netherlands	Nynke Rots, Gerco den Hartog, Rob van Binnendijk
Department of Nephrology, Leiden University Medical Center, Leiden, The Netherlands	Aiko P.J. de Vries

*CI is also affiliated with Department of Internal Medicine, Division of Nephrology, University of Groningen, University Medical Center Groningen, Groningen, the Netherlands.

Supplementary Table 1. Baseline characteristics of participants per study cohort

Characteristic	Control (N=42)	CKD (N=37)	Dialysis (N=38)	KTR (N=63)	p-value
Gender, no. (%)					<0.01[2]
Male	16 (38)	25 (68)	27 (71)	31 (49)	
Female	26 (62)	12 (32)	11 (29)	32 (51)	
Age at time of first dose, (IQR) – yr	61.5 (53.0 to 71.8)	65 (59.0 to 72.0)	63.5 (52.5 to 69.8)	61 (48.0 to 71.0)	0.48[1]
BMI, (IQR) - kg/m^2	27.9 (23.2 to 30.6)	27.5 (24.4 to 30.6)	25.4 (24.4 to 30.7)	26.1 (23.7 to 30.3)	0.74[1]
eGFR, (IQR) - mL/min/1.73m^2	75.7 (65.3 to 92.3)	18.1 (14.4 to 22.9)	-	45.5 (33.5 to 59.1)	<0.01[1]
Leukocyte count, (IQR) - 10^9/L	7.5 (6.6 to 14.4)	7.6 (6.4 to 9.2)	7.6 (6.0 to 9.4)	7.6 (6.0 to 9.4)	0.74[1]
Lymphocyte count, (IQR) - 10^9/L	2.3 (2.0 to 2.7)	1.6 (1.3 to 2.2)	1.4 (1.0 to 1.9)	1.5 (1.1 to 1.9)	<0.01[1]
Primary renal diagnosis, no. (%)					
Primary glomerulonephritis	-	1 (2.7)	2 (5.3)	13 (20.6)	
Interstitial nephritis	-	1 (2.7)	0 (0)	0 (0)	
Familial/hereditary renal diseases	-	1 (2.7)	5 (13.2)	9 (14.3)	
Congenital diseases	-	0 (0)	1 (2.6)	5 (7.9)	
Vascular diseases	-	14 (37.8)	9 (23.7)	12 (19.0)	
Secondary glomerular/systemic disease	-	0 (0)	1 (2.6)	0 (0)	
Diabetic kidney disease	-	5 (13.5)	7 (18.4)	5 (7.9)	
Other	-	12 (32.4)	9 (23.7)	10 (15.9)	
Dialysis characteristics					
Hemodialysis, no. (%)	-	-	27 (71)	-	
Peritoneal dialysis, no. (%)	-	-	11 (29)	-	
Time on dialysis, (IQR) – mo	-	-	23 (6 to 43)	-	
Transplant characteristics					
First kidney transplant, no. (%)	-	-	-	52 (81.3)	
Time after last transplantation, (IQR) – yr	-	-	-	6 (1 to 11)	
Last transplant					
Living, no. (%)	-	-	-	41 (65.1)	
Number of immunosuppressive agents, (IQR)	-	-	-	2 (2 to 2)	
Immunosuppressive treatment, no. (%)					
Steroids	-	-	-	20 (31.7)	
Azathioprine	-	-	-	2 (3.2)	
Mycophenolate mofetil	-	-	-	50 (79.4)	
Calcineurin inhibitor	-	-	-	60 (95.2)	
mTOR inhibitor	-	-	-	1 (1.6)	
Other	-	-	-	2 (3.2)	

Values are number (percentage) for categorical variables and median [interquartile range] for continuous variables.

[1]p-value based on non-parametric test (kruskal-wallis) test.
[2]p-value based on fisher's exact test.
BMI, body mass index; CKD, chronic kidney disease; KTR, kidney transplant recipient; eGFR, estimated glomerular filtration rate; mTOR, mammalian target of rapamycin; IL, interleukin; mo, month; yr, year.

9

Supplementary Table 2. Immune responses of participants per study cohort

	Total (N=180)	Control (N=42)	CKD (N=37)	Dialysis (N=38)	KTR (N=63)	p-value
Immune response pre vaccination						
S- specific binding antibodies, (IQR) - BAU/ml*	-0.47 (-1.00 to 0.10)	-0.39 (-1.00 to 0.13)	-0.24 (-1.00 to 0.16)	-0.86 (-1.00 to 0.06)	-0.41 (-1.00 to 0.03)	0.64[1]
IL-2, (IQR) - pg/ml*	-1.21 (-2.00 to 0.30)	- 0.24 (-2.00 to 0.55)	-0.91 (-2.00 to 0.11)	-2.00 (-2.00 to -0.06)	-2.00 (-2.00 to 0.24)	0.24[1]
IL-4, (IQR) - pg/ml*	-2.00 (-2.00 to 0.24)	-2.00 (-2.00 to 0.17)	-2.00 (-2.00 to 0.14)	-2.00 (-2.00 to -2.00)	-2.00 (-2.00 to 0.73)	0.41[1]
IL-5, (IQR) - pg/ml*	-2.00 (-2.00 to -0.15)	-2.00 (-2.00 to -2.00)	-2.00 (-2.00 to -2.00)	-2.00 (-2.00 to -0.47)	-2.00 (-2.00 to 0.46)	0.42[1]
IL-6, (IQR) - pg/ml*	3.36 (2.42 to 3.82)	3.48 (-0.95 to 3.78)	3.22 (2.42 to 3.8)	3.45 (2.21 to 3.92)	3.32 (2.77 to 3.83)	0.83[1]
IL-9, (IQR) - pg/ml*	-2.00 (-2.00 to 0.27)	-2.00 (-2.00 to 0.15)	-2.00 (-2.00 to -0.01)	-2.00 (-2.00 to 0.38)	-2.00 (-2.00 to 0.39)	0.92[1]
IL-10, (IQR) - pg/ml*	0.06 (-2.00 to 0.62)	-0.35 (-2.00 to0.46)	0.08 (-2.00 to 0.81)	0.04 (-2.00 to 0.39)	0.29 (-2.00 to 0.78)	0.09[1]
IL-13, (IQR) - pg/ml*	--2.00 (-2.00 to -2.00)	-2.00 (-2.00 to -2.00)	-2.00 (-2.00 to -2.00)	-2.00 (-2.00 to -2.00)	-2.00 (-2.00 to -2.00)	0.60[1]
IL-17A, (IQR) - pg/ml*	-2.00 (-2.00 to -0.45)	-2.00 (-2.00 to -0.46)	-2.00 (-2.00 to -0.45)	-2.00 (-2.00 to -1.72)	-2.00 (-2.00 to -0.40)	0.11[1]
IL-17F, (IQR) - pg/ml*	-2.00 (-2.00 to -0.72)	-2.00 (-2.00 to -2.00)	-2.00 (-2.00 to -2.00)	-2.00 (-2.00 to -1.00)	-2.00 (-2.00 to 0.43)	0.80[1]
IL-22, (IQR) - pg/ml*	-2.00 (-2.00 to 0.35)	-2.00 (-2.00 to 0.24)	-2.00 (-2.00 to0.18)	-2.00 (-2.00 to 0.13)	-2.00 (-2.00 to 0.85)	0.67[1]
TNF-α, (IQR) - pg/ml*	0.61 (-2.00 to 2.11)	0.47 (-2.00 to 2.23)	1.57 (-2.00 to 2.16)	-2.00 (-2.00 to 1.35)	0.80 (-2.00 to 2.11)	0.24[1]
IFN-γ, (IQR) - pg/ml*	-2.00 (-2.00 to 1.01)	-2.00 (-2.00 to 0.72)	0.37 (-2.00 to 0.99)	-2.00 (-2.00 to 0.86)	-2.00 (-2.00 to 1.12)	0.16[1]
Immune response on day 28 after second vaccination						
S-specific binding antibodies, (IQR) – BAU/ml*	3.09 (1.64 to 3.45)	3.41 (3.22 to 3.61)	3.24 (3.06 to 3.68)	3.21 (2.84 to 3.49)	0.79 (0.14 to 2.13)	<0.01[1]
Neutralizing antibodies, (IQR) – IU/ml*	2.42 (1.44 to 2.85)	2.78 (2.56 to 3.02)	2.77 (2.42 to 2.98)	2.43 (2.00 to 2.81)	0.00 (0.00 to 1.82)	<0.01[1]
IL-2, (IQR) - pg/ml*	2.13 (1.32 to 2.50)	2.46 (2.18 to 2.63)	2.24 (2.01 to 2.52)	2.24 (1.87 to 2.54)	1.16 (-0.03 to 1.68)	<0.01[1]
IL-4, (IQR) - pg/ml*	-2.00 (-2.00 to 0.78)	-0.02 (-2.00 to 0.77)	-2.00 (-2.00 to 0.77)	0.60 (-2.00 to 1.11)	-2.00 (-2.00 to 0.60)	0.06[1]
IL-5, (IQR) - pg/ml*	1.14 (-2.00 to 1.78)	1.52 (0.55 to 2.00)	1.37 (-0.22 to 1.80)	1.53 (-1.44 to 2.04)	-2.00 (-2.00 to 1.33)	<0.01[1]
IL-6, (IQR) - pg/ml*	2.99 (-2.00 to 3.60)	-0.85 (-2.00 to 3.14)	2.70 (-2.00 to 3.40)	3.35 (2.44 to 4.00)	3.30 (-2.00 to 3.72)	<0.01[1]
IL-9, (IQR) - pg/ml*	-2.00 (-2.00 to 0.51)	-2.00 (-2.00 to 0.49)	-2.00 (-2.00 to 0.53)	-0.09 (-2.00 to 0.69)	-2.00 (-2.00 to 0.09)	0.12[1]
IL-10, (IQR) - pg/ml*	0.25 (-2.00 to 0.82)	-0.14 (-2.00 to 0.59)	-2.00 (-2.00 to 0.53)	0.39 (-2.00 to 0.84)	0.55 (-2.00 to 0.88)	0.02[1]
IL-13, (IQR) - pg/ml*	0.14 (-2.00 to 1.50)	1.05 (-2.00 to 1.81)	0.96 (-2.00 to 1.61)	1.16 (-2.00 to 1.87)	-2.00 (-2.00 to 0.89)	<0.01[1]
IL-17A, (IQR) - pg/ml*	-2.00 (-2.00 to -0.21)	-2.00 (-2.00 to -0.04)	-2.00 (-2.00 to -0.28)	-2.00 (-2.00 to 0.06)	-2.00 (-2.00 to -0.52)	0.27[1]
IL-17F, (IQR) - pg/ml*	-2.00 (-2.00 to -0.07)	-2.00 (-2.00 to 0.47)	-2.00 (-2.00 to -2.00)	-2.00 (-2.00 to 0.03)	-2.00 (-2.00 to -0.13)	0.13[1]
IL-22, (IQR) - pg/ml*	-2.00 (-2.00 to 0.51)	-2.00 (-2.00 to 0.32)	-2.00 (-2.00 to 0.26)	-2.00 (-2.00 to 0.67)	-2.00 (-2.00 to 0.55)	0.68[1]

TNF-α, (IQR) - pg/ml*	1.29 (-2.00 to 2.25)	-0.65 (-2.00 to 1.72)	0.71 (-2.00 to 1.97)	2.31 (-2.00 to 2.53)	0.39 (-2.00 to 2.14)	<0.01[1]
IFN-γ, (IQR) - pg/ml*	1.97 (1.07 to 2.44)	2.43 (2.13 to 2.70)	2.20 (1.50 to 2.38)	2.37 (1.46 to 2.57)	1.11 (-2.00 to 1.67)	<0.01[1]
Immune response 6 months after second vaccination						
S-specific binding antibodies, (IQR) – BAU/ml*	2.17 (1.28 to 2.61)	2.62 (2.25 to 2.84)	2.48 (2.25 to 2.79)	2.07 (1.84 to 2.46)	0.87 (-0.20 to 1.85)	<0.01[1]
Neutralizing antibodies, (IQR) – IU/ml*	2.02 (1.48 to 2.51)	2.34 (2.01 to 2.70)	2.21 (1.72 to 2.54)	2.08 (1.59 to 2.40)	1.00 (0.00 to 2.16)	<0.01[1]

*median log 10 transformed SARS-CoV-2 specific immune response.
[1]p-value based on non-parametric test (kruskal-wallis) test.
IL, interleukin.

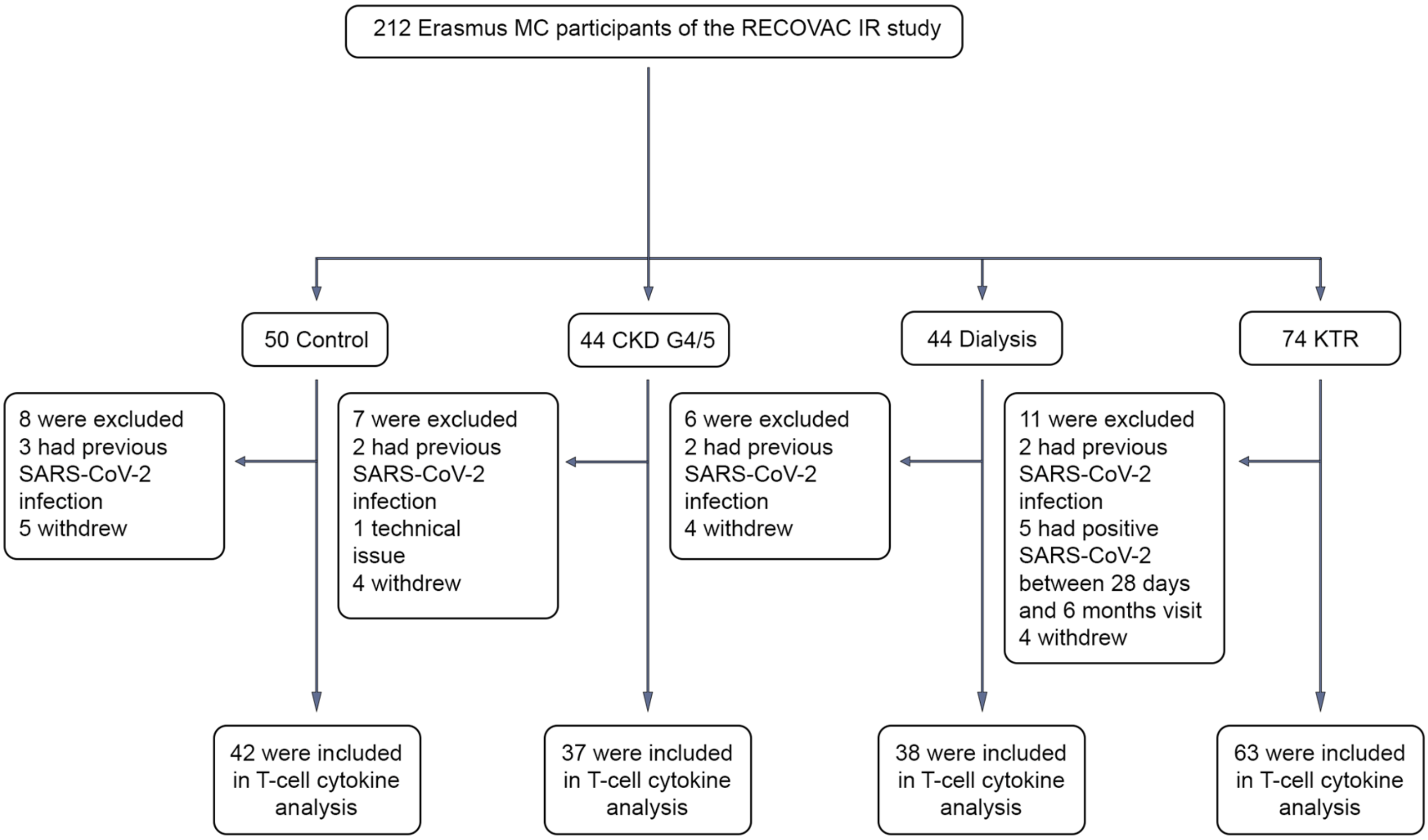

Supplementary Figure 1. Participant enrollment and analysis. CKD denotes chronic kidney disease; KTR denotes kidney transplant recipient; SARS-CoV-2 denotes severe acute respiratory syndrome coronavirus 2.

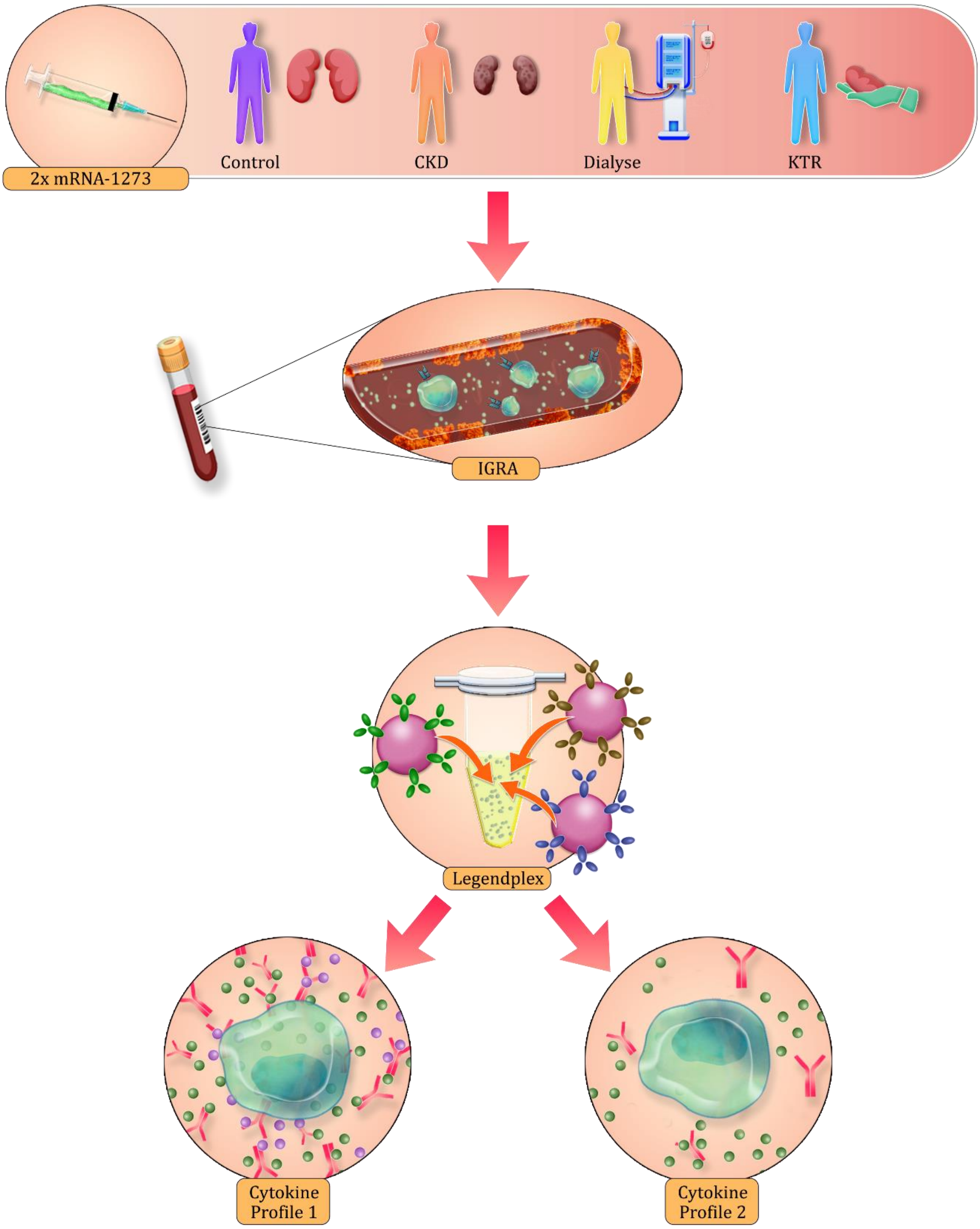

Supplementary Figure 2. Infographic about the study.

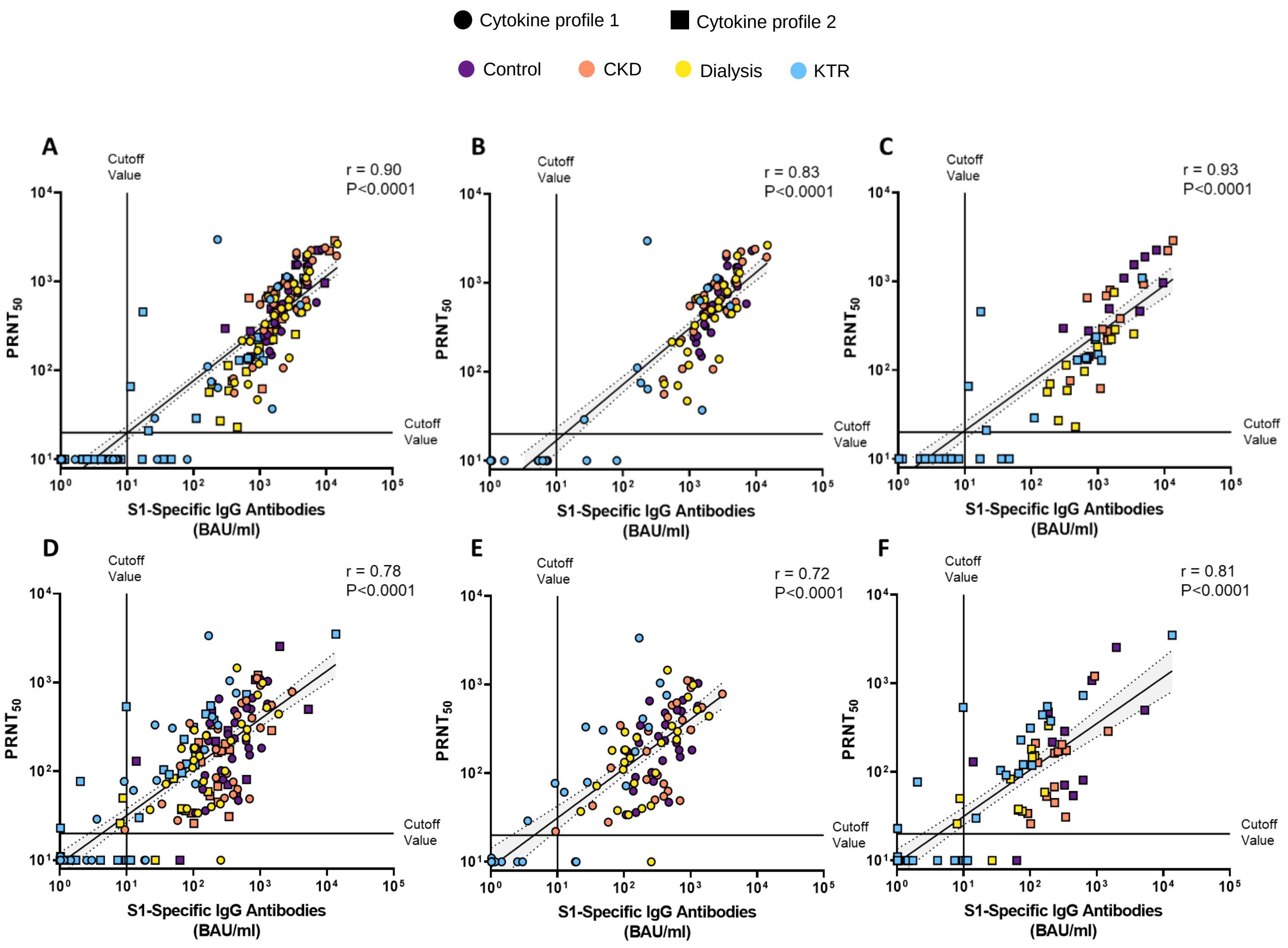

Supplementary Figure 3. Correlation between S1-specifc IgG antibodies and neutralizing antibodies.
A) Profile 1 and 2 S1-specifc IgG antibody levels correlated with neutralizing antibody levels, 28 days after second vaccination (Spearman's rank correlation coefficient 0.90; p<0.0001). Diagonal line represents the regression line on $\log_{10}$-transformed data (beta coefficient 0.59; 95% CI 0.55 to 0.63). B) Profile 1 Spearman's rank correlation coefficient 0.83; p<0.0001, beta coefficient 0.63; 95% CI 0.57 to 0.70. C) Profile 2 Spearman's rank correlation coefficient 0.93; p<0.0001, beta coefficient 0.55; 95% CI 0.49 to 0.60. D) Profile 1 and 2 S1-specific IgG antibody levels correlated with neutralizing antibody levels, 6 months after second vaccination (Spearman's rank correlation coefficient profile: 0.78; p<0.0001). Diagonal line represents the regression line on $\log_{10}$-transformed data (beta coefficient: 0.54; 95% CI 0.48 to 0.60). E) Profile 1 Spearman's rank correlation coefficient profile: 0.72; p<0.0001, beta coefficient: 0.56; 95% CI 0.46 to 0.65. F) Profile 2 Spearman's rank correlation coefficient profile: 0.81; p<0.0001, beta coefficient: 0.52; 95% CI 0.44 to 0.61. 95% CI limits of the best-fit line are presented as dotted lines. Each symbol in represents a participant.

16

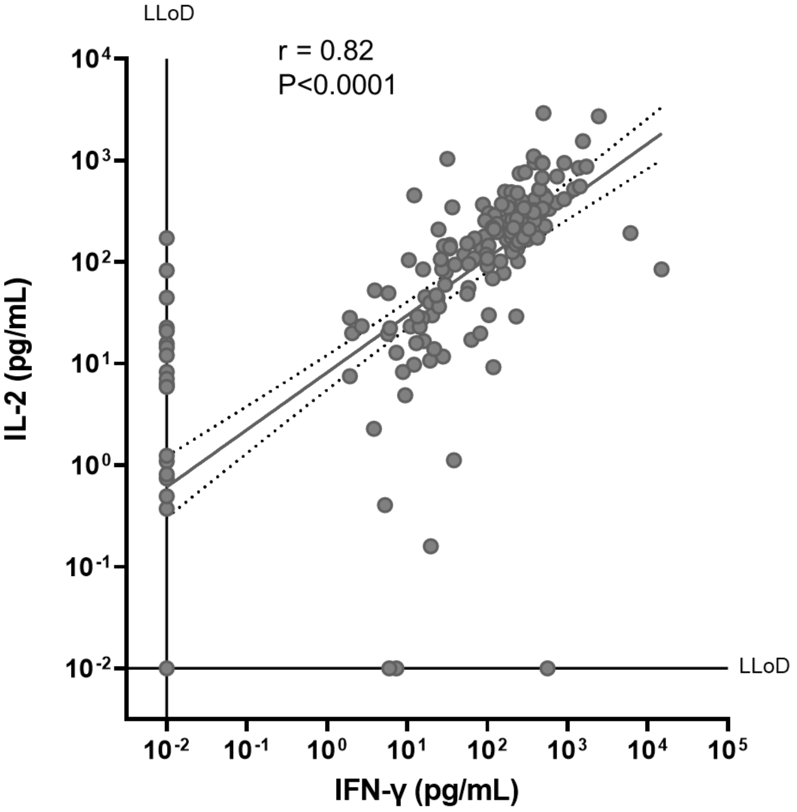

Supplementary Figure 4. Correlation between the Th_1 cytokines IFN-γ and IL-2. IFN-γ levels correlated with IL-2 levels (Spearman's rank correlation coefficient 0.82, p<0.0001). Diagonal line represents the regression line on log_{10}-transformed data (beta coefficient 0.56; 95% CI 0.48 to 0.64). 95% CI limits of the best-fit line are presented as dotted lines. Each symbol represents a participant. LLoD, lower limit of detection.

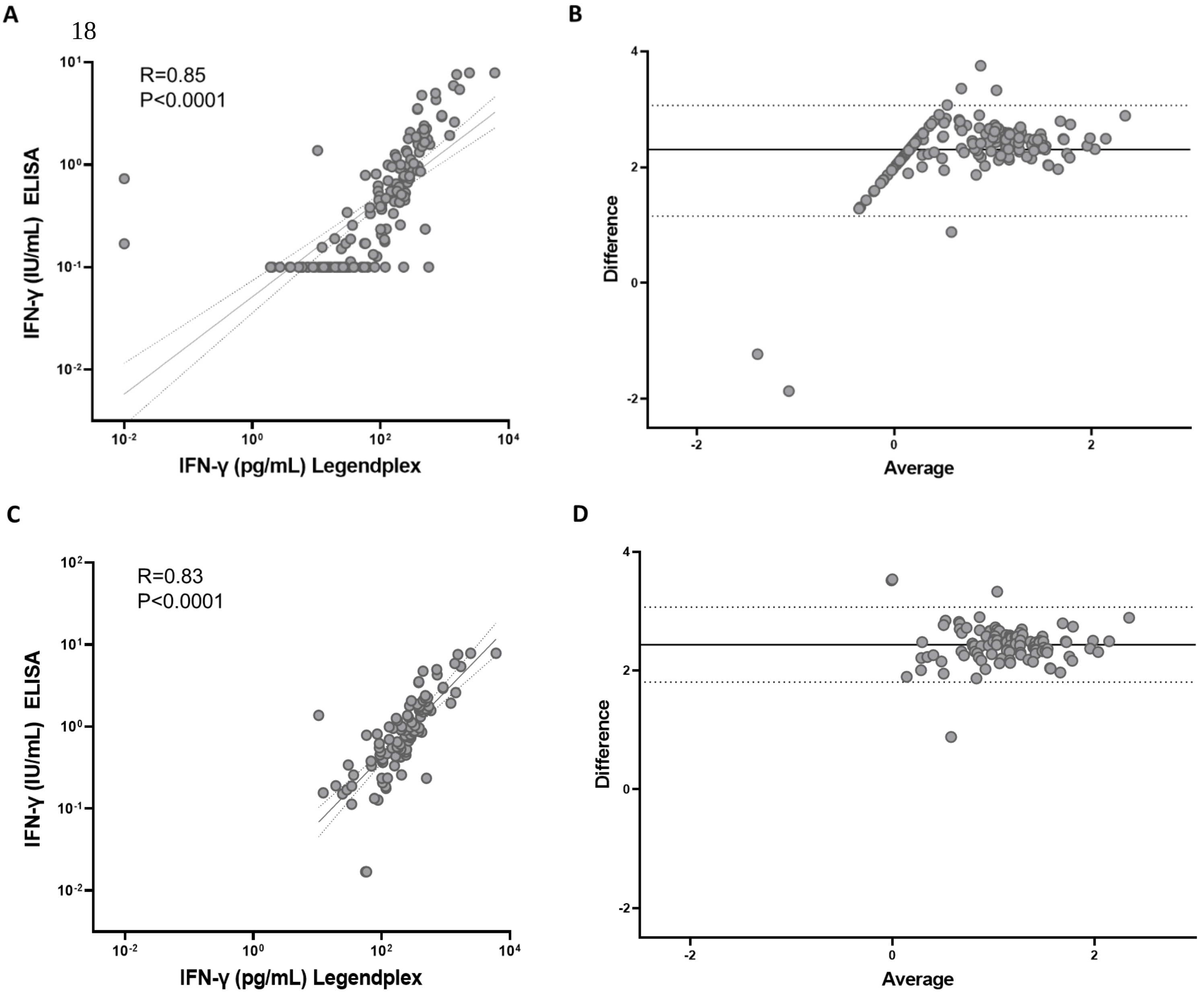

Supplementary Figure 5. Correlation and agreement of Legendplex and ELISA SARS-CoV-2 specific IFN-γ measurements after *ex vivo* stimulation. A) Legendplex measured IFN-γ levels correlated with ELISA measured IFN-γ levels, 28 days after second vaccination (Spearman's rank correlation coefficient 0.85; p<0.0001). Diagonal line represents the regression line on log$_{10}$-transformed responder in Legendplex and/or ELISA (n=154) data (beta coefficient 0.48; 95% CI 0.40 to 0.55). The 95% CI limits of the best-fit line are presented as dotted lines. B) Legendplex and ELISA IFN-γ levels are in agreement however Legendplex gives higher values, 28 days after second vaccination. Limits of agreement of log$_{10}$-transformed responder in Legendplex and/or ELISA (n=154) data are presented as dotted lines, bias (2.30) is presented as continuous line. C) Legendplex measured IFN-γ levels correlated with ELISA measured IFN-γ levels, 28 days after second vaccination (Spearman's rank correlation coefficient 0.83; p<0.001). Diagonal continuous line represents the regression line on log$_{10}$-transformed responder in both Legendplex and ELISA (n=104) data (beta coefficient 0.81; 95% CI 0.68 to 0.94). The 95% CI limits of the best-fit line are presented as dotted lines. D) Legendplex and ELISA IFN-γ levels are in agreement however Legendplex gives higher values, 28 days after second vaccination. Limits of agreement of log$_{10}$-transformed responder in both Legendplex and ELISA (n=104) data are presented as dotted lines, bias (2.44) is presented as continuous line. Each symbol represents a participant.
