# Supplementary material for: Th1-dominant cytokine responses in kidney patients after COVID-19 vaccination are associated with poor humoral responses
Source: NPJ Vaccines. 2023 May 17;8:70. doi: 10.1038/s41541-023-00664-4 (PMC10191401; doi:10.1038/s41541-023-00664-4)
Supplement: Supplementary file 2 — REPORTING SUMMARY [file 41541_2023_664_MOESM2_ESM.pdf]

nature portfolio

Corresponding author(s): Carla C. Baan

Last updated by author(s): Mar 13, 2023

Reporting Summary

Nature Portfolio wishes to improve the reproducibility of the work that we publish. This form provides structure for consistency and transparency in reporting. For further information on Nature Portfolio policies, see our Editorial Policies and the Editorial Policy Checklist.

Statistics

For all statistical analyses, confirm that the following items are present in the figure legend, table legend, main text, or Methods section.

n/a	Confirmed	
☐	☒	The exact sample size (n) for each experimental group/condition, given as a discrete number and unit of measurement
☒	☐	A statement on whether measurements were taken from distinct samples or whether the same sample was measured repeatedly
☐	☒	The statistical test(s) used AND whether they are one- or two-sided<br>*Only common tests should be described solely by name; describe more complex techniques in the Methods section.*
☐	☒	A description of all covariates tested
☐	☒	A description of any assumptions or corrections, such as tests of normality and adjustment for multiple comparisons
☐	☒	A full description of the statistical parameters including central tendency (e.g. means) or other basic estimates (e.g. regression coefficient) AND variation (e.g. standard deviation) or associated estimates of uncertainty (e.g. confidence intervals)
☒	☐	For null hypothesis testing, the test statistic (e.g. F, t, r) with confidence intervals, effect sizes, degrees of freedom and P value noted<br>*Give P values as exact values whenever suitable.*
☒	☐	For Bayesian analysis, information on the choice of priors and Markov chain Monte Carlo settings
☐	☒	For hierarchical and complex designs, identification of the appropriate level for tests and full reporting of outcomes
☒	☐	Estimates of effect sizes (e.g. Cohen's d, Pearson's r), indicating how they were calculated

Our web collection on statistics for biologists contains articles on many of the points above.

Software and code

Policy information about availability of computer code

Data collection	Cytokine data was collected with LEGENDplex V8.0 software
Data analysis	Statistical analyses were carried out with GraphPad Prism software version 9.1.2, and Rstudio software version 4.0.5.

For manuscripts utilizing custom algorithms or software that are central to the research but not yet described in published literature, software must be made available to editors and reviewers. We strongly encourage code deposition in a community repository (e.g. GitHub). See the Nature Portfolio guidelines for submitting code & software for further information.

Data

Policy information about availability of data

All manuscripts must include a data availability statement. This statement should provide the following information, where applicable:

- Accession codes, unique identifiers, or web links for publicly available datasets
- A description of any restrictions on data availability
- For clinical datasets or third party data, please ensure that the statement adheres to our policy

The data and R code that support the findings of this study are available from the corresponding author upon reasonable request.

Human research participants

Policy information about studies involving human research participants and Sex and Gender in Research.

Reporting on sex and gender	Findings in this study only apply to sex (biological trait) that were self-reported. We did not include sex and gender analysis as it was not relevant to this study.
Population characteristics	Four different cohorts were included; cohort A: participants without kidney disease (eGFR >45 mL/min/1.73m2); cohort B: patients with CKD stage G4/5 (eGFR <30 mL/min/1.73m2); cohort C: patients undergoing hemo- or peritoneal dialysis; and cohort D: Kidney transplant recipients.
Recruitment	From all participants who participated in the RECOVAC IR study we measured cytokines in the participants that were enrolled in Erasmus MC Rotterdam
Ethics oversight	The Dutch Central Committee on Research Involving Human Subjects (CCMO, NL76215.042.21) and the institutional review board of the Erasmus MC Rotterdam (MEC2020-662) approved the study

Note that full information on the approval of the study protocol must also be provided in the manuscript.

Field-specific reporting

Please select the one below that is the best fit for your research. If you are not sure, read the appropriate sections before making your selection.

☒ Life sciences ☐ Behavioural & social sciences ☐ Ecological, evolutionary & environmental sciences

For a reference copy of the document with all sections, see nature.com/documents/nr-reporting-summary-flat.pdf

Life sciences study design

All studies must disclose on these points even when the disclosure is negative.

Sample size	No sample-size calculation was performed, sample size was established by including the Erasmus MC Rotterdam participants of the RECOVAC IR study (n=180)
Data exclusions	There was no data excluded during this study from the analyses.
Replication	In this study we verify the reproducibility of our study by comparing the IFN-y concentrations of the same samples measured by both Legendplex and ELISA via a Spearman's correlation. We found that the two techniques are highly correlated (Supplemental Figure S5).
Randomization	Allocation was not random because we were intrested in the SARS-CoV-2 specific repsonses in the different patient groups.
Blinding	Blinding was not relevant in our study because all participants received mRNA-1273 vaccination.

Reporting for specific materials, systems and methods

We require information from authors about some types of materials, experimental systems and methods used in many studies. Here, indicate whether each material, system or method listed is relevant to your study. If you are not sure if a list item applies to your research, read the appropriate section before selecting a response.

Materials & experimental systems

n/a	Involved in the study	
☐	☒	Antibodies
☐	☒	Eukaryotic cell lines
☒	☐	Palaeontology and archaeology
☒	☐	Animals and other organisms
☒	☐	Clinical data
☒	☐	Dual use research of concern

Methods

n/a	Involved in the study	
☒	☐	ChIP-seq
☒	☐	Flow cytometry
☒	☐	MRI-based neuroimaging

Antibodies

Antibodies used	polyclonal rabbit anti–SARS-CoV-2 nucleocapsid antibody (Sino Biological; 40143-V08B) and a secondary peroxidase-labeled goat anti-rabbit IgG (Dako; P0448).
Validation	The rabbit polyclonal anti-SARS-CoV-2 nucleocapsid antibody has been validated for ELISA using corresponding virus-positive samples (stated on the manufacturer's website). This antibody is used in combination with polyclonal goat anti-rabbit immunoglobulins/HRP that have been validated in a direct sandwich ELISA procedure (stated on the manufacturer's website). In addition, the combination of these two antibodies is used in other publications.<br>1) Geers D, Shamier MC, Bogers S, den Hartog G, Gommers L, Nieuwkoop NN, Schmitz KS, Rijsbergen LC, van Osch JAT, Dijkhuizen E, Smits G, Comvalius A, van Mourik D, Caniels TG, van Gils MJ, Sanders RW, Oude Munnink BB, Molenkamp R, de Jager HJ, Haagmans BL, de Swart RL, Koopmans MPG, van Binnendijk RS, de Vries RD, GeurtsvanKessel CH. SARS-CoV-2 variants of concern partially escape humoral but not T-cell responses in COVID-19 convalescent donors and vaccinees. Sci Immunol. 2021 May 25;6(59):eabj1750. doi: 10.1126/sciimmunol.abj1750. PMID: 34035118; PMCID: PMC9268159.<br><br>2) Okba NMA, Müller MA, Li W, Wang C, GeurtsvanKessel CH, Corman VM, Lamers MM, Sikkema RS, de Bruin E, Chandler FD, Yazdanpanah Y, Le Hingrat Q, Descamps D, Houhou-Fidouh N, Reusken CBEM, Bosch BJ, Drosten C, Koopmans MPG, Haagmans BL. Severe Acute Respiratory Syndrome Coronavirus 2-Specific Antibody Responses in Coronavirus Disease Patients. Emerg Infect Dis. 2020 Jul;26(7):1478-1488. doi: 10.3201/eid2607.200841. Epub 2020 Jun 21. PMID: 32267220; PMCID: PMC7323511.

Eukaryotic cell lines

Policy information about cell lines and Sex and Gender in Research

Cell line source(s)	The VeroE6 cells are originally from ATCC
Authentication	As confiremed by ATCC
Mycoplasma contamination	The cells are periodically tested for mycoplasma contamination. If they are negative, we use them for experiments.
Commonly misidentified lines<br>(See ICLAC register)	x
